# Supplementary material for: Public sector’s efficiency as a reflection of governance quality, an European Union study
Source: PLoS One. 2023 Sep 8;18(9):e0291048. doi: 10.1371/journal.pone.0291048 (PMC10490916; doi:10.1371/journal.pone.0291048)
Supplement: S1 Fig — Source: authors’ processing. (DOCX) [file pone.0291048.s001.docx]

**S1 Fig. Histogram of the efficiency scores**

Source: authors’ processing
