# Supplementary figures and images for: Public sector’s efficiency as a reflection of governance quality, an European Union study
Source: PLoS One. 2023 Sep 8;18(9):e0291048. doi: 10.1371/journal.pone.0291048 (PMC10490916; doi:10.1371/journal.pone.0291048)

**
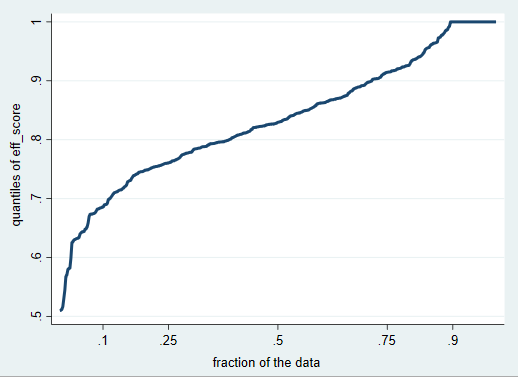
S2 Fig**. **Quantile distribution of the dependent variable *eff_score***

Source: authors’ processing

Supplement: S2 Fig — Source: authors’ processing. (DOCX) [file pone.0291048.s002.docx]
