# Supplementary material for: Public sector’s efficiency as a reflection of governance quality, an European Union study
Source: PLoS One. 2023 Sep 8;18(9):e0291048. doi: 10.1371/journal.pone.0291048 (PMC10490916; doi:10.1371/journal.pone.0291048)
Supplement: S1 Table — Data source: authors’ processing. (DOCX) [file pone.0291048.s003.docx]

**S1 Table. Efficiency scores of the EU27 countries in terms of good governance and public efficiency, obtained using DEA methodology**

|  |  | 2005 | 2006 | 2007 | 2008 | 2009 | 2010 | 2011 | 2012 | 2013 | 2014 | 2015 | 2016 | 2017 | 2018 | 2019 | 2020 |
| --- | --- | --- | --- | --- | --- | --- | --- | --- | --- | --- | --- | --- | --- | --- | --- | --- | --- |
| 1 | **Austria** | 0.7857 | 0.7927 | 0.7847 | 0.7856 | 0.7885 | 0.7873 | 0.8062 | 0.7776 | 0.761 | 0.7704 | 0.7953 | 0.8233 | 0.7963 | 0.8278 | 0.8266 | 0.8044 |
| 2 | **Belgium** | 0.9035 | 0.9103 | 0.9145 | 0.9255 | 0.8924 | 0.8975 | 0.8738 | 0.8616 | 0.8528 | 0.8678 | 0.8816 | 0.9037 | 0.918 | 0.9554 | 0.9562 | 0.9209 |
| 3 | **Bulgaria** | 0.8407 | 0.9142 | 0.8689 | 0.9091 | 0.9256 | 0.9907 | 1 | 0.9565 | 1 | 1 | 1 | 1 | 0.9816 | 0.9795 | 1 | 1 |
| 4 | **Croatia** | 1 | 1 | 1 | 1 | 0.9847 | 0.9999 | 0.9638 | 0.9288 | 0.9118 | 0.9028 | 0.9382 | 0.917 | 0.8981 | 0.9408 | 0.9776 | 0.9259 |
| 5 | **Cyprus** | 0.915 | 0.8825 | 0.8989 | 0.8704 | 0.8919 | 0.8878 | 0.8863 | 0.8608 | 0.8628 | 0.8117 | 0.8625 | 0.8959 | 0.8875 | 0.9774 | 0.9656 | 1 |
| 6 | **Czech Republic** | 0.6435 | 0.644 | 0.6735 | 0.6696 | 0.6748 | 0.6857 | 0.6829 | 0.6894 | 0.69 | 0.674 | 0.6762 | 0.7148 | 0.7058 | 0.7449 | 0.7687 | 0.7308 |
| 7 | **Denmark** | 0.8629 | 0.8709 | 0.8497 | 0.8481 | 0.8252 | 0.8297 | 0.8404 | 0.8412 | 0.8387 | 0.8262 | 0.8308 | 0.8498 | 0.8573 | 0.866 | 0.8621 | 0.8536 |
| 8 | **Estonia** | 0.7529 | 0.723 | 0.7487 | 0.7394 | 0.7421 | 0.754 | 0.7668 | 0.7457 | 0.7212 | 0.6825 | 0.7105 | 0.7143 | 0.7114 | 0.7495 | 0.7538 | 0.7179 |
| 9 | **Finland** | 0.7963 | 0.8032 | 0.7881 | 0.7937 | 0.7641 | 0.7617 | 0.7641 | 0.7596 | 0.76 | 0.7481 | 0.7485 | 0.7564 | 0.758 | 0.7574 | 0.7599 | 0.7553 |
| 10 | **France** | 0.7786 | 0.764 | 0.7764 | 0.7831 | 0.7908 | 0.7785 | 0.7861 | 0.7844 | 0.7933 | 0.7945 | 0.8225 | 0.8491 | 0.796 | 0.8499 | 0.8226 | 0.8223 |
| 11 | **Germany** | 0.7964 | 0.7784 | 0.8011 | 0.8135 | 0.8074 | 0.8206 | 0.8301 | 0.8205 | 0.7973 | 0.7562 | 0.7882 | 0.7997 | 0.7932 | 0.8174 | 0.8334 | 0.8139 |
| 12 | **Greece** | 0.7619 | 0.788 | 0.811 | 0.8446 | 0.9361 | 0.9729 | 0.9619 | 0.9741 | 0.9025 | 1 | 0.9862 | 1 | 1 | 1 | 1 | 0.8896 |
| 13 | **Hungary** | 0.5819 | 0.5795 | 0.6268 | 0.6735 | 0.7101 | 0.7369 | 0.7409 | 0.7545 | 0.7469 | 0.785 | 0.7947 | 0.8449 | 0.7771 | 0.845 | 0.8752 | 0.8266 |
| 14 | **Ireland** | 0.7284 | 0.7392 | 0.7417 | 0.7304 | 0.7456 | 0.6988 | 0.7517 | 0.7589 | 0.7602 | 0.7344 | 0.8258 | 0.8638 | 0.87 | 0.9171 | 0.9545 | 0.9153 |
| 15 | **Italy** | 0.8899 | 0.8993 | 0.9408 | 0.9367 | 0.937 | 0.9483 | 0.9531 | 0.9346 | 0.9176 | 0.92 | 0.9326 | 0.9207 | 0.9138 | 0.9639 | 0.96 | 0.8916 |
| 16 | **Latvia** | 0.8408 | 0.8146 | 0.8829 | 0.9215 | 0.8711 | 0.8343 | 0.8918 | 0.8649 | 0.8378 | 0.8076 | 0.8228 | 0.8238 | 0.8089 | 0.8678 | 0.8576 | 0.8354 |
| 17 | **Lithuania** | 0.7753 | 0.7731 | 0.7886 | 0.8035 | 0.7749 | 0.7772 | 0.7986 | 0.7744 | 0.7506 | 0.7484 | 0.7446 | 0.7458 | 0.7551 | 0.8005 | 0.7977 | 0.7545 |
| 18 | **Luxembourg** | 1 | 1 | 1 | 1 | 1 | 1 | 1 | 1 | 1 | 1 | 1 | 1 | 1 | 1 | 1 | 1 |
| 19 | **Malta** | 0.5166 | 0.5116 | 0.5099 | 0.5103 | 0.5304 | 0.5447 | 0.5704 | 0.5673 | 0.5814 | 0.5988 | 0.6252 | 0.6436 | 0.6339 | 0.6743 | 0.7524 | 0.6899 |
| 20 | **Netherlands** | 0.9034 | 0.9053 | 0.912 | 0.9232 | 0.8975 | 0.8944 | 0.8684 | 0.8546 | 0.8506 | 0.8348 | 0.8621 | 0.873 | 0.874 | 0.9061 | 0.9238 | 0.9041 |
| 21 | **Poland** | 0.6586 | 0.7033 | 0.682 | 0.6487 | 0.6323 | 0.6304 | 0.642 | 0.6326 | 0.6397 | 0.6298 | 0.648 | 0.7084 | 0.7116 | 0.7678 | 0.7841 | 0.7653 |
| 22 | **Portugal** | 0.7295 | 0.7908 | 0.8123 | 0.7959 | 0.7961 | 0.8276 | 0.8343 | 0.8207 | 0.8053 | 0.7985 | 0.7935 | 0.8082 | 0.7658 | 0.8116 | 0.8253 | 0.809 |
| 23 | **Romania** | 1 | 1 | 1 | 1 | 1 | 1 | 0.9733 | 1 | 0.9466 | 1 | 0.917 | 0.8894 | 1 | 1 | 0.9399 | 0.8607 |
| 24 | **Slovak Republic** | 0.6316 | 0.6517 | 0.6856 | 0.6787 | 0.6842 | 0.7008 | 0.7134 | 0.6992 | 0.692 | 0.6845 | 0.7147 | 0.72 | 0.7168 | 0.7804 | 0.8213 | 0.7928 |
| 25 | **Slovenia** | 0.8519 | 0.8463 | 0.8691 | 0.8673 | 0.872 | 0.9033 | 0.8859 | 0.889 | 0.8174 | 0.8268 | 0.8489 | 0.8308 | 0.8437 | 0.879 | 0.8793 | 0.87 |
| 26 | **Spain** | 0.8555 | 0.9649 | 0.9864 | 0.9925 | 1 | 1 | 0.9437 | 0.9243 | 0.9424 | 0.926 | 0.9352 | 0.9149 | 0.9232 | 0.9616 | 0.9643 | 0.92 |
| 27 | **Sweden** | 0.8655 | 0.8751 | 0.8677 | 0.8624 | 0.8292 | 0.8216 | 0.8428 | 0.8467 | 0.8452 | 0.8236 | 0.8218 | 0.8328 | 0.8261 | 0.8267 | 0.8097 | 0.8109 |
|  | **EU average** | 0.8025 | 0.8121 | 0.8230 | 0.8269 | 0.8261 | 0.8328 | 0.8360 | 0.8267 | 0.8157 | 0.8131 | 0.8269 | 0.8387 | 0.8342 | 0.8692 | 0.8767 | 0.8474 |
|  | **Max** | 1 | 1 | 1 | 1 | 1 | 1 | 1 | 1 | 1 | 1 | 1 | 1 | 1 | 1 | 1 | 1 |
|  | **Min** | 0.5166 | 0.5116 | 0.5099 | 0.5103 | 0.5304 | 0.5447 | 0.5704 | 0.5673 | 0.5814 | 0.5988 | 0.6252 | 0.6436 | 0.6339 | 0.6743 | 0.7524 | 0.6899 |
|  | **Std. Dev.** | 0.1234 | 0.1254 | 0.1215 | 0.1233 | 0.1199 | 0.1233 | 0.1107 | 0.1087 | 0.1038 | 0.1126 | 0.1026 | 0.0935 | 0.1003 | 0.0925 | 0.0845 | 0.0850 |
|  | **Old EU avg** | 0.8327 | 0.8487 | 0.8562 | 0.8597 | 0.8579 | 0.8599 | 0.8612 | 0.8542 | 0.8448 | 0.8429 | 0.8589 | 0.8711 | 0.8637 | 0.8901 | 0.8927 | 0.8651 |
|  | **New EU avg** | 0.7699 | 0.7726 | 0.7873 | 0.7917 | 0.7919 | 0.8035 | 0.8089 | 0.7972 | 0.7845 | 0.7809 | 0.7926 | 0.8037 | 0.8024 | 0.8467 | 0.8595 | 0.8284 |

Data source: authors’ processing
