# Supplementary material for: Public sector’s efficiency as a reflection of governance quality, an European Union study
Source: PLoS One. 2023 Sep 8;18(9):e0291048. doi: 10.1371/journal.pone.0291048 (PMC10490916; doi:10.1371/journal.pone.0291048)
Supplement: S2 Table — Data source: authors’ processing. (DOCX) [file pone.0291048.s004.docx]

**S2 Table. EU27 states’ rankings by efficiency scores obtained using DEA methodology**

|  | 2005 | 2006 | 2007 | 2008 | 2009 | 2010 | 2011 | 2012 | 2013 | 2014 | 2015 | 2016 | 2017 | 2018 | 2019 | 2020 |
| --- | --- | --- | --- | --- | --- | --- | --- | --- | --- | --- | --- | --- | --- | --- | --- | --- |
| Bulgaria | 13 | 5 | 11 | 9 | 7 | 5 | 1 | 4 | 1 | 1 | 1 | 1 | 4 | 4 | 1 | 1 |
| Cyprus | 4 | 9 | 8 | 10 | 10 | 11 | 9 | 11 | 8 | 13 | 9 | 8 | 9 | 5 | 5 | 1 |
| Luxembourg | 1 | 1 | 1 | 1 | 1 | 1 | 1 | 1 | 1 | 1 | 1 | 1 | 1 | 1 | 1 | 1 |
| Croatia | 1 | 1 | 1 | 1 | 4 | 4 | 4 | 6 | 6 | 7 | 4 | 5 | 8 | 9 | 4 | 4 |
| Belgium | 5 | 6 | 6 | 6 | 9 | 9 | 11 | 10 | 9 | 8 | 8 | 7 | 6 | 8 | 8 | 5 |
| Spain | 10 | 4 | 4 | 4 | 1 | 1 | 7 | 7 | 4 | 5 | 5 | 6 | 5 | 7 | 6 | 6 |
| Ireland | 22 | 21 | 22 | 22 | 21 | 24 | 22 | 21 | 19 | 22 | 13 | 11 | 11 | 10 | 9 | 7 |
| Netherlands | 6 | 7 | 7 | 7 | 8 | 10 | 12 | 12 | 10 | 9 | 10 | 10 | 10 | 11 | 11 | 8 |
| Italy | 7 | 8 | 5 | 5 | 5 | 7 | 6 | 5 | 5 | 6 | 6 | 4 | 7 | 6 | 7 | 9 |
| Greece | 19 | 17 | 15 | 14 | 6 | 6 | 5 | 3 | 7 | 1 | 3 | 1 | 1 | 1 | 1 | 10 |
| Slovenia | 11 | 12 | 10 | 11 | 11 | 8 | 10 | 8 | 14 | 10 | 11 | 16 | 13 | 12 | 12 | 11 |
| Romania | 1 | 1 | 1 | 1 | 1 | 1 | 3 | 1 | 3 | 1 | 7 | 9 | 1 | 1 | 10 | 12 |
| Denmark | 9 | 11 | 13 | 13 | 14 | 13 | 14 | 14 | 12 | 11 | 12 | 12 | 12 | 14 | 14 | 13 |
| Latvia | 12 | 13 | 9 | 8 | 12 | 12 | 8 | 9 | 13 | 14 | 14 | 17 | 15 | 13 | 15 | 14 |
| Hungary | 26 | 26 | 26 | 24 | 23 | 22 | 23 | 22 | 22 | 17 | 18 | 14 | 19 | 16 | 13 | 15 |
| France | 17 | 20 | 20 | 20 | 17 | 18 | 19 | 17 | 17 | 16 | 15 | 13 | 17 | 15 | 19 | 16 |
| Germany | 14 | 18 | 16 | 15 | 15 | 16 | 16 | 16 | 16 | 19 | 20 | 20 | 18 | 19 | 16 | 17 |
| Sweden | 8 | 10 | 12 | 12 | 13 | 15 | 13 | 13 | 11 | 12 | 16 | 15 | 14 | 18 | 21 | 18 |
| Portugal | 21 | 16 | 14 | 17 | 16 | 14 | 15 | 15 | 15 | 15 | 19 | 19 | 20 | 20 | 18 | 19 |
| Austria | 16 | 15 | 19 | 19 | 18 | 17 | 17 | 18 | 18 | 18 | 17 | 18 | 16 | 17 | 17 | 20 |
| Slovak Republic | 25 | 24 | 23 | 23 | 24 | 23 | 24 | 24 | 24 | 23 | 23 | 23 | 23 | 22 | 20 | 21 |
| Poland | 23 | 23 | 24 | 26 | 26 | 26 | 26 | 26 | 26 | 26 | 26 | 26 | 24 | 23 | 23 | 22 |
| Finland | 15 | 14 | 18 | 18 | 20 | 20 | 21 | 20 | 20 | 21 | 21 | 21 | 21 | 24 | 25 | 23 |
| Lithuania | 18 | 19 | 17 | 16 | 19 | 19 | 18 | 19 | 21 | 20 | 22 | 22 | 22 | 21 | 22 | 24 |
| Czech Republic | 24 | 25 | 25 | 25 | 25 | 25 | 25 | 25 | 25 | 25 | 25 | 24 | 26 | 26 | 24 | 25 |
| Estonia | 20 | 22 | 21 | 21 | 22 | 21 | 20 | 23 | 23 | 24 | 24 | 25 | 25 | 25 | 26 | 26 |
| Malta | 27 | 27 | 27 | 27 | 27 | 27 | 27 | 27 | 27 | 27 | 27 | 27 | 27 | 27 | 27 | 27 |

Data source: authors’ processing
