# Supplementary material for: Public sector’s efficiency as a reflection of governance quality, an European Union study
Source: PLoS One. 2023 Sep 8;18(9):e0291048. doi: 10.1371/journal.pone.0291048 (PMC10490916; doi:10.1371/journal.pone.0291048)
Supplement: S3 Table — Data source: authors’ processing. (DOCX) [file pone.0291048.s005.docx]

**S3 Table. Descriptive statistics for the EU 27 sample**

| **Variable** | **Obs.** | **Mean** | **Std. Dev.** | **Min** | **Max** |
| --- | --- | --- | --- | --- | --- |
| *eff_score* | 432 | 0.8318 | 0.1088 | 0.5099 | 1.0000 |
| *hdi* | 405 | 0.8741 | 0.0419 | 0.7580 | 0.9550 |
| *pop_density* | 405 | 171.0578 | 258.2397 | 17.2000 | 1595.1000 |
| *old_depr* | 432 | 26.3236 | 4.7475 | 15.1734 | 36.6261 |
| *migr* | 432 | 2.4938 | 6.9022 | -25.2000 | 40.4000 |
| *cpi_rescaled* | 432 | 3.6769 | 1.6487 | 0.4000 | 7.0000 |
| *demo_index* | 432 | 0.7402 | 0.0910 | 0.3446 | 0.8572 |
| *ec_freed* | 405 | 7.6861 | 0.3051 | 6.5500 | 8.3600 |
| *trade* | 432 | 124.4964 | 64.7408 | 45.4200 | 380.1000 |
| *fdi* | 432 | 13.7804 | 43.6376 | -57.6055 | 449.0828 |
| *e-gov* | 349 | 47.5989 | 19.9035 | 5.0000 | 92.0000 |

Data source: authors’ processing
