# Supplementary material for: Public sector’s efficiency as a reflection of governance quality, an European Union study
Source: PLoS One. 2023 Sep 8;18(9):e0291048. doi: 10.1371/journal.pone.0291048 (PMC10490916; doi:10.1371/journal.pone.0291048)
Supplement: S4 Table — Data source: authors’ processing. (DOCX) [file pone.0291048.s006.docx]

**S4 Table. Correlation matrix**

|  | *eff_score* | *hdi* | *pop_density* | *old_depr* | *migr* | *cpi_rescaled* | *demo_index* | *ec_freed* | *trade* | *fdi* | *egov* |
| --- | --- | --- | --- | --- | --- | --- | --- | --- | --- | --- | --- |
| *eff_score* | 1 |  |  |  |  |  |  |  |  |  |  |
| *hdi* | 0.0121 | 1 |  |  |  |  |  |  |  |  |  |
| *pop_density* | -0.3271 | 0.0985 | 1 |  |  |  |  |  |  |  |  |
| *old_depr* | 0.1957 | 0.2446 | -0.0209 | 1 |  |  |  |  |  |  |  |
| *migr* | -0.0008 | 0.3655 | 0.392 | -0.0569 | 1 |  |  |  |  |  |  |
| *cpi_rescaled* | 0.0576 | -0.7995 | -0.0389 | -0.0332 | -0.3155 | 1 |  |  |  |  |  |
| *demo_index* | -0.1193 | 0.6508 | -0.0841 | 0.0388 | 0.1505 | -0.6376 | 1 |  |  |  |  |
| *ec_freed* | -0.2048 | 0.4591 | 0.1107 | 0.0261 | 0.2184 | -0.5335 | 0.2415 | 1 |  |  |  |
| *trade* | -0.1684 | 0.1061 | 0.5256 | -0.3856 | 0.3567 | -0.126 | -0.0964 | 0.2357 | 1 |  |  |
| *fdi* | -0.1959 | -0.0799 | 0.3936 | -0.3204 | 0.0751 | -0.02 | -0.0979 | 0.0202 | 0.2818 | 1 |  |
| *egov* | -0.1065 | 0.7474 | -0.0003 | 0.2345 | 0.251 | -0.7724 | 0.4613 | 0.4492 | 0.1531 | -0.0981 | 1 |

Data source: authors’ processing
