# Supplementary material for: Public sector’s efficiency as a reflection of governance quality, an European Union study
Source: PLoS One. 2023 Sep 8;18(9):e0291048. doi: 10.1371/journal.pone.0291048 (PMC10490916; doi:10.1371/journal.pone.0291048)
Supplement: S5 Table — Standard errors in parentheses. *** p<0.01, ** p<0.05, * p<0.1. Source: authors’ processing. (DOCX) [file pone.0291048.s007.docx]

**S5 Table. OLS and panel data regression results**

| **Dependent variable: Efficiency score** | | | | | |
| --- | --- | --- | --- | --- | --- |
| **Independent variables** | **(1)** | **(2)** | **(3)** | **(4)** | **(5)** |
|  | **OLS** | **FEM** | **REM** | **LSDV** | **PCSE** |
|  |  |  |  |  |  |
| *hdi* | 0.6573836** | 0.2860888 | 0.6452061* | 0.2860888 | 0.6677643** |
|  | (0.2639707) | (0.3779570) | (0.3311799) | (0.3779570) | (0.3027817) |
| *pop_density* | -0.0002242*** | 0.0003957*** | 0.0000240 | 0.0003957*** | -0.0001505*** |
|  | (0.0000266) | (0.0001015) | (0.0000632) | (0.0001015) | (0.0000406) |
| *old_depr* | 0.0084928*** | 0.0008969 | 0.0016027 | 0.0008969 | 0.0040841** |
|  | (0.0016172) | (0.0017803) | (0.0016265) | (0.0017803) | (0.0019442) |
| *migr* | 0.0026985*** | 0.0015299*** | 0.0017104*** | 0.0015299*** | 0.0009026** |
|  | (0.0008959) | (0.0003927) | (0.0003918) | (0.0003927) | (0.0004443) |
| *cpi_rescaled* | -0.0073331 | 0.0072963 | 0.0093473* | 0.0072963 | 0.0095128 |
|  | (0.0071013) | (0.0051449) | (0.0050314) | (0.0051449) | (0.0062424) |
| *demo_index* | -0.2262060*** | -0.3508276*** | -0.3205617*** | -0.3508276*** | -0.2233579** |
|  | (0.0811273) | (0.0528132) | (0.0523373) | (0.0528132) | (0.0960394) |
| *ec_freed* | -0.0934984*** | -0.0741713*** | -0.0767379*** | -0.0741713*** | -0.0238196 |
|  | (0.0208762) | (0.0164367) | (0.0163309) | (0.0164367) | (0.0189705) |
| *trade* | 0.0003545*** | 0.0004742*** | 0.0001658 | 0.0004742*** | 0.0001508 |
|  | (0.0001227) | (0.0001719) | (0.0001501) | (0.0001719) | (0.0001463) |
| *fdi* | 0.0003853** | -0.0001273 | -0.0001751** | -0.0001273 | -0.0000248 |
|  | (0.0001725) | (0.0000813) | (0.0000819) | (0.0000813) | (0.0000726) |
| *egov* | -0.0016965*** | -0.0000464 | -0.0002101 | -0.0000464 | -0.0001109 |
|  | (0.0004919) | (0.0002962) | (0.0002971) | (0.0002962) | (0.0003237) |
| Constant | 1.0066663*** | 1.2355423*** | 1.0021718*** | 1.2496111*** | 0.4691310* |
|  | (0.2798297) | (0.2863299) | (0.2616742) | (0.2964364) | (0.2845204) |
|  |  |  |  |  |  |
| Observations | 323 | 323 | 323 | 323 | 323 |
| R-squared | 0.2943657 | 0.4095719 |  | 0.9326666 | 0.8827851 |
| F Statistic |  | 27 | 27 |  | 27 |
| P value | 0.0000 | 0.0000 | 0.0000 | 0.0000 | 0.0000 |
| Breusch-Pagan / Cook-Weisberg test for heteroskedasticity | 1.03 |  |  |  |  |
| White test | 196.82 |  |  |  |  |
| Test parm | 23.90 |  |  |  |  |
| Breusch–Pagan Lagrangian multiplier test |  |  | 1248.57 |  |  |
| Hausman test |  | 17.98 |  |  |  |
| Sargan Hansen |  | 61.529 |  |  |  |
| Wald test |  | 748.57 |  |  |  |
| Pesaran test |  | 11.393 |  |  |  |
| Wooldridge test |  | 26.607 |  |  |  |

Standard errors in parentheses

*** p<0.01, ** p<0.05, * p<0.1

Source: authors’ processing
