# Supplementary material for: Public sector’s efficiency as a reflection of governance quality, an European Union study
Source: PLoS One. 2023 Sep 8;18(9):e0291048. doi: 10.1371/journal.pone.0291048 (PMC10490916; doi:10.1371/journal.pone.0291048)
Supplement: S6 Table — Standard errors in parentheses. *** p<0.01, ** p<0.05, * p<0.1. Source: authors’ processing. (DOCX) [file pone.0291048.s008.docx]

**S6 Table. Heteroskedasticity robust standard errors quantile regression results**

| **Dependent variable: Efficiency score** | | | | | |
| --- | --- | --- | --- | --- | --- |
| **Independent variables** | **(1)** | **(2)** | **(3)** | **(4)** | **(5)** |
|  | **Q10** | **Q25** | **Q50** | **Q75** | **Q90** |
|  |  |  |  |  |  |
| *hdi* | 0.5131192 | 0.7406806** | 1.1531351*** | 1.1425130** | 0.9646113* |
|  | (0.6179221) | (0.3598035) | (0.3101397) | (0.4810909) | (0.5380419) |
| *pop_density* | -0.0001847** | -0.0001598*** | -0.0002303*** | -0.0001389 | -0.0000676 |
|  | (0.0000795) | (0.0000373) | (0.0000716) | (0.0001077) | (0.0000680) |
| *old_depr* | 0.0111568*** | 0.0075765*** | 0.0074199** | 0.0010947 | -0.0017034 |
|  | (0.0042488) | (0.0018176) | (0.0031240) | (0.0026587) | (0.0018968) |
| *migr* | 0.0025407 | 0.0016335* | 0.0027860* | 0.0025896*** | 0.0019919 |
|  | (0.0022891) | (0.0009438) | (0.0014195) | (0.0009971) | (0.0014157) |
| *cpi_rescaled* | 0.0067721 | 0.0004842 | 0.0104314 | 0.0147451* | 0.0140144** |
|  | (0.0199819) | (0.0139358) | (0.0094781) | (0.0085626) | (0.0060912) |
| *demo_index* | -0.0933311 | -0.1377786 | -0.3006210 | -0.4262651*** | -0.4192185*** |
|  | (0.1183606) | (0.0862317) | (0.2129573) | (0.1357400) | (0.1372528) |
| *ec_freed* | -0.0655574 | -0.0875125** | -0.1007380*** | -0.0932569*** | -0.0901969*** |
|  | (0.0595912) | (0.0410921) | (0.0309092) | (0.0193704) | (0.0182679) |
| *trade* | 0.0002683 | -0.0000972 | 0.0000470 | 0.0004033*** | 0.0002897** |
|  | (0.0004545) | (0.0002649) | (0.0004699) | (0.0001401) | (0.0001255) |
| *fdi* | 0.0005286 | 0.0004920*** | 0.0004403** | -0.0001455 | -0.0001669 |
|  | (0.0011686) | (0.0001409) | (0.0001817) | (0.0001609) | (0.0003412) |
| *egov* | -0.0003945 | -0.0013127** | -0.0019909*** | -0.0006101 | -0.0004751 |
|  | (0.0010656) | (0.0005435) | (0.0007361) | (0.0006998) | (0.0004957) |
| Constant | 0.5200242 | 0.7874399 | 0.6871970** | 0.8572628** | 1.0936586*** |
|  | (0.6944269) | (0.5814165) | (0.3198862) | (0.3650282) | (0.3693877) |
|  |  |  |  |  |  |
| Observations | 323 | 323 | 323 | 323 | 323 |
| R-squared | 0.2369142 | 0.2387001 | 0.2533013 | 0.1406741 | 0.0643938 |

Standard errors in parentheses

*** p<0.01, ** p<0.05, * p<0.1

Source: authors’ processing
